# Mating Success, Longevity, and Fertility of *Diabrotica virgifera virgifera* LeConte (Chrysomelidae: Coleoptera) in Relation to Body Size and Cry3Bb1-Resistant and Cry3Bb1-Susceptible Genotypes

**DOI:** 10.3390/insects6040943

**Published:** 2015-11-10

**Authors:** Bryan Wade French, Leslie Hammack, Douglas W. Tallamy

**Affiliations:** 1 North Central Agricultural Research Laboratory (NCARL), USDA-ARS, 2923 Medary Ave., Brookings, SD 57006, USA; 2 NCARL, USDA-ARS, 13786 Thomas Place, Keystone, SD 57751, USA; E-Mail: hammackl@mt-rushmore.net; 3 Department of Entomology and Wildlife Ecology, University of Delaware, Newark, DE 19716, USA; E-Mail: dtallamy@udel.edu

**Keywords:** mating frequency, courtship duration, copulation duration, life history traits, leaf beetle, western corn rootworm, transgenic maize

## Abstract

Insect resistance to population control methodologies is a widespread problem. The development of effective resistance management programs is often dependent on detailed knowledge regarding the biology of individual species and changes in that biology associated with resistance evolution. This study examined the reproductive behavior and biology of western corn rootworm beetles of known body size from lines resistant and susceptible to the Cry3Bb1 protein toxin expressed in transgenic *Bacillus thuringiensis* maize. In crosses between, and within, the resistant and susceptible genotypes, no differences occurred in mating frequency, copulation duration, courtship duration, or fertility; however, females mated with resistant males showed reduced longevity. Body size did not vary with genotype. Larger males and females were not more likely to mate than smaller males and females, but larger females laid more eggs. Moderately strong, positive correlation occurred between the body sizes of successfully mated males and females; however, weak correlation also existed for pairs that did not mate. Our study provided only limited evidence for fitness costs associated with the Cry3Bb1-resistant genotype that might reduce the persistence in populations of the resistant genotype but provided additional evidence for size-based, assortative mating, which could favor the persistence of resistant genotypes affecting body size.

## 1. Introduction

The western corn rootworm, *Diabrotica virgifera virgifera* LeConte, is an important economic pest of maize, *Zea mays* L., in the United States and more recently in Europe, with an estimated one billion dollars in annual control costs in the U.S. alone [1,2,3]. This insect has repeatedly demonstrated its resilience in overcoming population control attempts by evolving physiological resistance to insecticides [4,5,6,7] and behavioral resistance to crop rotation involving increased oviposition in non-maize fields where hatching larvae may survive if maize is grown the following season [8]. In subsequent attempts to control corn rootworm populations, the United States Environmental Protection Agency approved the first of many genetically modified maize hybrids in 2003 [9,10]. These modified hybrids produce one or two rootworm-toxic proteins originating from the common soil bacterium *Bacillus thuringiensis* Berliner (*Bt*) and have offered a novel technological advance to manage corn rootworm populations [11,12].

Prior to approving the registration of *Bt* transgenic crops, however, the USEPA [9] required seed companies developing these hybrids to submit and subsequently implement an insect resistance management plan. The plans were intended to hinder resistance evolution to the *Bt* toxins. Contingent on the number of toxins produced by the hybrids, commercial growers planting a *Bt* transgenic maize that targets corn rootworms must also plant a portion (5%–20%) of their maize acreage to a non-*Bt* hybrid, which must be planted either adjacent to or within the *Bt* field. The purpose of this non-*Bt* refuge is to provide susceptible corn rootworm beetles able to disperse from the refuge and mate with any resistant individuals emerging from the *Bt* plants, thus slowing resistance evolution to the *Bt* toxins and lengthening the effective lifespan of the *Bt* varieties.

Laboratory studies have demonstrated the importance of non-*Bt* refuges in that resistance to single *Bt* toxins can quickly evolve when beetles surviving *Bt* exposure cannot mate with beetles unexposed to the *Bt* hybrid, especially in the case of the lower-dose varieties historically used for managing corn rootworm populations [13,14,15,16,17,18]. This model of mating restriction also may have contributed to the current widespread *Bt* resistance discovered in *D. v. virgifera* field populations [19,20,21]. Given the rapid development of resistance, there is a commonly accepted necessity for fundamental research on corn rootworm biology, encompassing mating behavior, dispersal, and other fitness related traits, to support the development of insect resistance management tactics that promote the mating of surviving *Bt*-exposed beetles with unexposed susceptible beetles [9,22,23,24,25].

In addition to mating restrictions, evolving resistance to *Bt* crops and other management options may be connected to fitness costs associated with traits such as survival, developmental time, fecundity, longevity, body size, and mating behavior [26,27,28]. However, because the single *Bt* toxins used to control corn rootworm populations are considered a low to moderate dose of *Bt* toxin compared with those in the maize varieties used for lepidopteran control [12,14,17,29,30], few to no significant fitness costs have been linked with corn rootworm survival on single-toxin-producing *Bt* maize [16,31,32,33,34]. Nevertheless, the *Bt* maize events expressing higher-dose toxins for corn rootworm control (e.g., pyramid toxins produced by SmartStax^®^ varieties) theoretically could have fitness costs associated with resistance evolution.

Male and female insects usually differ in reproductive strategies due, partly, to differences in life history strategies [35,36]. The choice by a female to mate with a particular male could be contingent on direct resources (e.g., nuptial gifts and territory for feeding and/or oviposition) that the male provides the female prior to or during copulation and/or on indirect benefits, such as relatively superior genes that may enhance the fitness of her offspring [37,38,39,40]. A female may prefer to mate with a larger male because of his provision of both direct (more resources) and indirect benefits (superior genes) if those benefits enhance her fitness. In many insect species, body size is directly related to fitness [35,39,41], and large females generally lay more eggs than smaller females, as reported for *D. v. virgifera* [42].

Our primary goal was to examine certain aspects of the mating behavior and reproductive biology of *D. v. virgifera* beetles of known body size in relation to resistance and susceptibility to the Cry3Bb1 protein toxin expressed in maize [11]. Here, we report for *Bt*-susceptible and *Bt*-resistant lines and for reciprocal no-choice crosses between the two genotypes the mating success of mixed-sex pairs as well as the courtship and copulation durations, longevity, and fecundity of the females that mated. Mating success and female fecundity were also examined in relation to body size, as was the correlation by mating status between the body sizes of the males and females composing the mixed-sex pairs.

## 2. Materials and Methods

### 2.1. Resistant and Susceptible Populations

A genetically diverse line of non-diapausing *D. v. virgifera* was developed at the USDA, ARS, North Central Agricultural Research Laboratory in Brookings, South Dakota, by crossing females from a non-diapausing laboratory colony with males from diapausing colonies established from four geographically distinct field populations [18]. These beetles were reared as larvae on maize seedlings using a protocol similar to that described by Jackson [43], as were all generations (F0–F29). From the genetically diverse non-diapausing colony, three Cry3Bb1-resistant sub-lines were incrementally selected for eleven generations: F0–F10 [18]. Additionally, two susceptible or control sub-lines were reared similarly to the selected lines but without exposure to the Cry3Bb1 toxin. These sub-lines of the starting colonies (one susceptible and one resistant) were reared in successive weeks to provide a steady supply of insects. We used beetles equally from all lines as there were no fitness differences during selection [18]. Because pesticide and Cry3Bb1 resistance that carries few fitness costs persists for some generations in the absence of selection [14,34,44], all the lines were reared on non-transgenic maize beginning with F12. All the beetles used in the present study were from F25–F29.

### 2.2. Handling of Experimental Insects

Pupal weight is strongly correlated with adult body weight in field-collected and laboratory-reared *D. v. virgifera*, and the pupal weight served as a measure of body size for the current experiments (Table A1). We obtained pupae reared as described in the previous paragraph by carefully excavating them from their plastic rearing containers (340 × 250 × 99 mm) and pupal cells, assigned them a unique identification number, and then determined their sex based on the presence/absence of papillae on the venter near the apex of the abdomen [45] at 25× magnification using a stereo microscope (Wild M32, Heerbrugg, Switzerland). Next, we weighed the pupae to the nearest 0.01 mg using an analytical electronic balance (OHAUS AP250D, Florham Park, NJ, USA). Pupae were housed individually in a 7-mL plastic cup (Nalgene, Rochester, NY, USA) on the surface of approximately 5-mL of dried and sifted 80-mesh (177-μm) soil moistened with distilled water. We subsequently placed the pupae in an environmental chamber at 25 °C and 60% RH in darkness and checked daily for eclosion.

Within one day of eclosion, but after hardening of the cuticle, each adult was transferred to an individual plastic cage containing fresh artificial diet and a cotton-plugged vial of water. The food was changed once or twice weekly and was identical to that developed by Branson and Jackson [46], except for the substitution of fructose for sucrose and the addition of the antibiotics spectinomycin and lincomycin to control mycoplasma and bacteria. Each cage was constructed of a 6.5-cm-diameter, 0.17-liter container glued at its bottom to the upper surface of a 15-cm-diameter lid attached to a 15-cm-diameter, 1.15-liter container. To provide air circulation, approximately 5-cm-diameter holes were drilled above one another through the lids of both containers and the bottom of the smaller container. A plastic screen mesh (8 strands/cm) covered the hole in the 6.5-cm lid. The containers were transferred to a small holding room, where the insects were maintained at 25 °C and 60% RH under a 14 h light:10 h dark photoperiod until their use in the mating trials.

### 2.3. Mating and Female Fertility Experiments

We grouped beetles identified by individual number, sex (M = male; F = female), and genotype (S = susceptible; R = resistant) in 10- × 35-mm Petri dishes into the following mixed-sex pairs: SMSF, SMRF, RMSF, and RMRF, with each pair assigned a unique number. The mean body weight of these beetles as pupae and their mean age at pairing, as well as variability measures for both parameters, are summarized by sex and genotype in Table 1. All the pairs were observed for at least two hours. Copulations that began within two hours were observed until the sexes separated, except in one case of extended copulation. Our aim was to observe approximately 30 mated pairs for each mating arrangement. The pre-copulation or courtship duration was logged as the time elapsed between mixing the sexes and the beginning of copulation. We considered copulation to start when the entire aedeagus of the male appeared to be inserted into the female reproductive tract and his hind legs were situated completely off of the substrate and positioned at the base of the female abdomen near her genitalia. Copulation was considered complete when the male and female separated and terminated sexual interactions. Despite the occurrence of cryptic female choice leading to the prevention of spermatophore transfer in certain *Diabrotica* beetles [47,48], the aforementioned visual observation method for determining a successful copulation, that is, the complete transfer of a spermatophore, proved 100% effective in earlier studies of *D. v. virgifera* calling behavior [49,50] and *Diabrotica barberi* Smith and Lawrence reproduction in relation to body size [51].

All the males, and the females that did not mate within two hours, were preserved in 95% ethanol. The females that mated successfully were returned to their individual containers in the holding room. After one week, an oviposition dish (15- × 60-mm Petri dish) that was filled to a depth of approximately 4 mm with soil was added to each container. The soil had been sifted through an 80-mesh (177-μm) screen and was thoroughly moistened with distilled water. We drilled a small hole approximately 5 mm in diameter into the Petri dish lid, allowing the female access to the dish for oviposition, and covered the lid with a small, accordion-shaped piece of sheet metal to deter light entry and facilitate oviposition. We replaced the egg dishes weekly until each female died and labeled the dishes with the date of female entry and removal, as well as the numbers of the female, the mating pair, and the dish. We stored the egg dishes in a cold room at 8 °C until the eggs could be separated from the soil by washing the soil through a 60-mesh (250-μm) sieve to collect the eggs [52]. The total number of eggs was tallied while simultaneously counting those with visually discernible physical damage or discoloration, with potentially viable or good eggs showing no visually discernible physical damage or discoloration [53].

**Table 1:** Mean pupal weights (mg) and ages (days) at time of pairing by sex and genotype of *D. v. virgifera* adults used in the mating experiments. Genotype is determined by resistance or susceptibility to the *Bt* Cry3Bb1 toxin.

Sex	Genotype	*n*	Weight ± SD	Range	Age ± SD	Range
Male	Resistant	138	11.4 ± 1.8	6.6–17.1	6.9 ± 2.6	2–14
	Susceptible	151	11.8 ± 1.8	7.4–16.6	7.5 ± 3.2	3–18
Female	Resistant	140	12.6 ± 2.2	7.7–18.9	5.2 ± 2.7	1–11
	Susceptible	149	12.8 ± 2.0	8.3–17.3	6.7 ± 3.3	1–16

### 2.4. Statistical Analysis

Our statistical procedures followed Zar [54] or the techniques provided in the Statistical Analysis System software [55]. We assessed data normality with visual methods, including qq probability plots, and formal tests of normality, including those of Shapiro Wilk, Anderson-Darling, Cramer-von Mises, and Kolmogorov-Smirnov, using PROC UNIVARIATE [54,55]. If the raw data did not meet parametric assumptions, we then performed a log (*x* + 1) data transformation. Non-parametric tests were used when the assumption of data normality or normally distributed residuals was deemed inappropriate.

For each sex, comparisons between the genotypes were made using unpaired *t*-tests for pupal weights and Mann-Whitney *U* tests for adult ages. Contingency chi-square analyses examined the frequency of mating success among the four combinations of male and female genotypes (2 × 4) and between the two male genotypes after pooling data across female genotypes (2 × 2). For the successfully mated pairs, a Kruskal-Wallis *H* test and Mann-Whitney *U* test were performed to evaluate the differences in courtship duration among the four combinations of male and female genotypes and between male genotypes after pooling data from the female genotypes, respectively. A two-way analysis of variance (ANOVA) evaluated the effect on copulation duration of male and female genotypes, the main effects in the model, and of the interaction between male and female genotypes. We also examined differences in female longevity and fecundity among the four combinations of successfully mated pairs by Kruskal-Wallis *H* tests and, after combining data from the resistant and susceptible mated females, between females with resistant and susceptible male mating partners by Mann-Whitney *U* tests. The fecundity data were restricted to weeks one through 12 of egg collection and derived only from those females that lived for two weeks or more after pairing. This 12-week interval approximates the maximum oviposition period under field conditions in the upper Midwest [51].

Unpaired *t*-tests were used to compare body weights between mated and unmated individuals by sex after pooling data across genotypes. Pearson’s correlation coefficient (*r*) was calculated to evaluate the strength of association between male and female body sizes for both the successfully mated pairs and those that did not mate, with Fisher’s z transformation applied to calculate 95% confidence intervals (CIs) around the *r* values. The relationship between the fecundity of mated females and their body size was evaluated using Spearman rank-order correlation and Fisher’s z transformation to obtain the 95% CI for the correlation coefficient.

## 3. Results

For the insects used in this study (Table 1), there was no difference in mean pupal weight between the resistant and susceptible males (*t* = −1.8; df = 287; *p* = 0.073) or between the resistant and susceptible females (*t* = −0.6; df = 287; *p* = 0.550). There was also no difference in age at mating between the resistant and susceptible males (Mann-Whitney *U* = 9371; *p* = 0.140); however, age did differ between the resistant and susceptible females (Mann-Whitney *U* = 7682; *p* = 0.0001).

In the 289 mating trials, 120 females (59 resistant and 61 susceptible) mated successfully, and 169 females (81 resistant and 88 susceptible) did not mate (Table 2), despite the occurrence of male mating attempts, with the male mounting the female in 75% of the unsuccessful cases. We found no differences in the frequency of mating success among the four combinations of male and female genotypes (*χ^2^* = 3.2; df = 3; *p* = 0.364) or between the resistant and susceptible males after pooling the data across female genotypes (*χ^2^* = 0.005; df = 1; *p* = 0.943).

**Table 2:** Proportion of pairs mating (%), courtship durations, and copulatory durations for successful copulations in crosses between Cry3Bb1-resistant (R) and Cry3Bb1-susceptible (S) genotypes of male (M) and female (F) *D. v. virgifera*.

Cross	Proportion Mating (%)	Duration (min)			
		Courtship		Copulation	
		Mean ± SD	Range	Mean ± SD	Range
SMSF	33/89 (37)	14.7 ± 22.6	1–103	174.6 ± 30.0	103–231
SMRF	30/62 (48)	11.6 ± 16.9	0–83	179.7 ± 31.4	127–292
RMSF	28/60 (47)	16.8 ± 30.9	1–118	160.4 ± 31.3	110–223
RMRF	29/78 (37)	18.7 ± 23.4	1–79	176.4 ± 36.4	120–270

The courtship and copulation durations for the successfully mating males and females are depicted in Table 2. We found no significant differences in courtship duration among the four crosses (Kruskal-Wallis *H* = 2.65; df = 3; *p* = 0.449) or between the male genotypes (RM = 17.8 ± 27.1 min with *n* = 57 and SM = 13.2 ± 20.0 min with *n* = 63) after pooling data across the female genotypes (Mann-Whitney *U* = 1922; *p* = 0.506). Similarly, after log (*x* + 1) data transformation, no significant differences occurred for copulation duration attributable to male genotype (*F* = 2.75; df = 1, 114; *p* = 0.100), female genotype (*F* = 3.32; df = 1, 114; *p* = 0.071), or the interaction between male and female genotypes (*F* = 0.86; df = 1, 114; *p* = 0.356). The sample size for the analysis of copulation duration was smaller than that for the courtship duration by two females because the observation of one pair was accidently terminated prematurely and the copulation duration of another pair was not recorded because separation occurred sometime after more than seven hours of observation.

After excluding two females that escaped captivity and one that was accidently killed, no significant difference in longevity occurred among mated females from the four mating combinations (Kruskal-Wallis *H* = 7.030; df = 3; *p* = 0.071) (Table 3). However, a comparison of longevity between females mated to either a resistant or a susceptible male indicated that the lifespan of females mated to resistant males was shorter than that of females mated to susceptible males (Mann-Whitney *U* = 1334; *p* = 0.0413) (Table 4).

**Table 3:** Longevity (days) for successfully mated females and numbers of eggs oviposited during 12 weeks beginning one week after mating for females living two weeks or more by cross between Cry3Bb1-resistant (R) and Cry3Bb1-susceptible (S) genotypes of male (M) and female (F) *D. v. virgifera*.

Cross	*n*	Longevity *		Total Eggs ^†^		Potentially Viable Eggs ^‡^	
		Mean ± SD	Range	Mean ± SD	Range	Mean ± SD	Range
SMSF	32	133 ± 45	22–191	1860 ± 789	68–3092	1780 ± 766	68–3024
SMRF	29	139 ± 47	26–210	1728 ± 871	1–3187	1646 ± 850	0–3142
RMSF	27	130 ± 51	12–220	1885 ± 701	122–3014	1822 ± 707	105–2980
RMRF	29	106 ± 57	7–241	1686 ± 851	387–3398	1610 ± 835	382–3380

* *n* = 117 after one female was accidently killed and two females escaped captivity; **^†^** Number of females living two weeks or more for SMSF, SMRF, RMSF, and RMRF crosses was 33, 29, 26, and 25, respectively; **^‡^** Total eggs less damaged and discolored eggs.

In addition to the omission of the female that was accidently killed, six females that died of natural causes prior to receiving their second egg dish were excluded from the oviposition analyses. The range of egg numbers varied greatly, with some females laying over 3000 eggs (Table 3). No statistically significant difference in the number of eggs laid over the 12-week period was detected among the females from the four crosses (Kruskal-Wallis *H* = 1.390; df = 3; *p* = 0.708) or between those females mated to a resistant compared with a susceptible male (Mann-Whitney *U* = 1533; *p* = 0.7840) (Table 4).

**Table 4:** Longevity in days, total eggs laid, and potentially viable eggs laid for *D. v. virgifera* females mated to a Cry3Bb1-resistant or Cry3Bb1-susceptible male.

Male	Longevity *		Total Eggs		Potentially Viable Eggs ^†^	
	*n*	Mean ± SD	*n* ^‡^	Mean ± SD	*n*	Mean ± SD
Resistant	56	117 ± 55 ^a^	51	1787 ± 777	51	1718 ± 772
Susceptible	61	136 ± 45 ^b^	62	1798 ± 825	62	1717 ± 802

* Means followed by different letters differ significantly by the Mann-Whitney *U* test (*p* < 0.05); **^†^** Total eggs less damaged and discolored eggs; **^‡^** Number of females living two weeks or more.

The number of potentially viable eggs laid per female during the 12-week oviposition period also varied greatly (Table 3), without a statistically significant difference in egg production among the females from the four crosses (Kruskal-Wallis *H* = 1.346; df = 3; *p* = 0.718). Potentially viable egg production also did not vary significantly between females mated to either a resistant male or a susceptible male (Mann-Whitney *U* = 1548; *p* = 0.8490) (Table 4).

**Figure 1:**
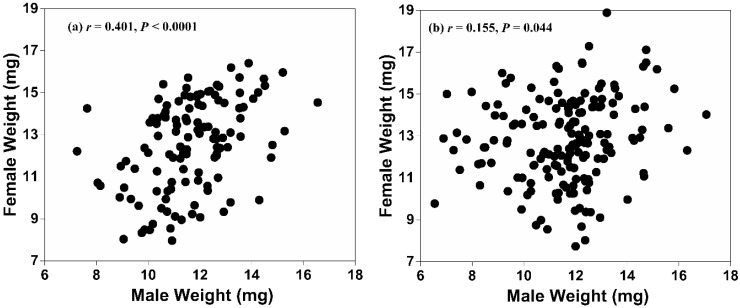
Scattergram showing the relationship between the pupal weights of males and females in pairs that (**a**) copulated successfully and (**b**) those that did not copulate.

After pooling weights across genotypes by sex, we found no weight differences between either males that mated successfully and those that did not (11.6 ± 1.6 mg with *n* = 120 and 11.6 ± 1.9 mg with *n* = 169, respectively; *t* = −0.1; df = 287; *p* = 0.907) or females that mated successfully and those that did not (12.5 ± 2.1 mg with *n* = 120 and 12.8 ± 2.0 mg with *n* = 169, respectively; *t* = 1.0; df = 287; *p* = 0.311). Using data pooled from the four combinations of male and female genotypes, an analysis of the relationship between the body sizes of the paired individuals showed a moderate-strength, positive correlation for successfully mated pairs between their male and female pupal weights (*r* = 0.401, 95% CI 0.237 to 0.540; *p* < 0.0001). However, a weaker correlation between male and female pupal weights also occurred in a similar analysis conducted with the pairs that did not mate (*r* = 0.155, 95% CI 0.004 to 0.299; *p* = 0.044) (Figure 1). For each of these analyses and the following one regarding fecundity, the data were pooled after first establishing overlap of the 95% CIs around the *r* values calculated for each of the four genotype combinations.

**Figure 2:**
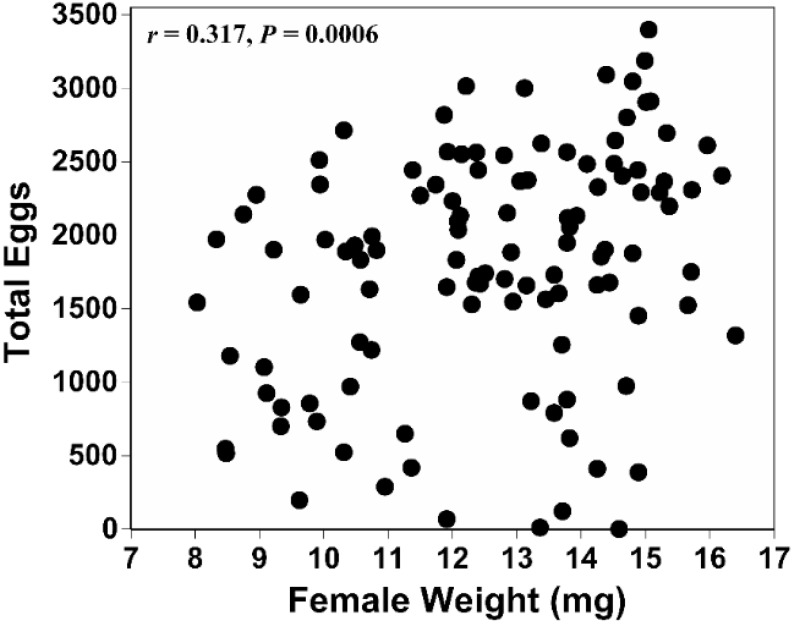
Scattergram showing the relationship between the pupal weight of mated females and their fecundity measured during a 12-week oviposition period.

After pooling the fecundity data for the females from the four crosses, the total number of eggs laid was correlated with the body size of the mated females (*r* = 0.317; 95% CI 0.139 to 0.473; *p =* 0.0006) (Figure 2).

## 4. Discussion

Our study provided little evidence for an influence of the Cry3Bb1-resistant genotype on the no-choice mating behavior and reproductive biology of *D. v. virgifera*. Whether the Cry3Bb1-resistant genotype was associated with one or both sexes in mixed-sex pairs established with virgin insects, we detected no effect of resistance on the mating success of the pairs or on the courtship and copulation durations of those pairs that mated successfully. The same was true for the fecundity of the mated females measured as total or viable numbers of eggs oviposited over a 12-week period; however, after pooling data from females of both genotypes, the females mated with Cry3Bb1-resistant males showed shorter longevity than that of the females mated with susceptible males. Most likely, there were several reasons that this apparent effect on longevity was not reflected in the fecundity data. First, the fecundity data were highly variable. Additionally, all of the females included in the longevity analysis but omitted from the egg analyses due to death during the first two weeks after mating had been mated with resistant males, and *D. v. virgifera* females oviposit most of their eggs later than the first two weeks after mating. If confirmed, the reduced longevity of females copulating with a Cry3Bb1-resistant male could reduce their fitness and thereby act to slow resistance evolution. In other words, reduced longevity could lead to reduced reproductive success through reduced fecundity, thereby reducing the persistence of resistant insects in populations and slowing resistance evolution. Using one line of females and multiple lines of males in *Drosophila melanogaster* (Meigen), the longevity of mated females was shown to vary considerably with the genotype of their mating partner, although the lower longevities in this case may have evolved as a consequence of multiple mating by females [56,57], which is uncommon in *D. v. virgifera* [58,59].

Several differences in the effects of resistance on fitness occurred between our study and a recent study of Hoffmann *et al.* [34], who used variant lines of the Cry3Bb1-resistant and Cry3Bb1-susceptible lines developed by Oswald *et al.* [18] and conducted their experiments one to seven generations after exposure to *Bt* maize. These authors detected greater adult longevity that was independent of sex for the resistant compared with the susceptible strain instead of the reduced adult longevity suggested by our study for females mated with males of the resistant strain. The greater longevity was also associated with increased fecundity in resistant females, a fitness benefit that was not detected in our study. This resistance benefit was, however, offset by a nearly 10% reduction in egg hatch rates. We found no effects of resistance on potential egg viability determined visually but did not directly measure egg hatch rates. The differences in fitness costs detected in our study compared with that of Hoffmann *et al.* [34] could derive from differences in the handling of the Cry3Bb1-resistant and Cry3Bb1-susceptible strains subsequent to their development by Oswald *et al.* [31], including differences in the number of generations elapsed between larval rearing on *Bt* maize and fitness testing. Additional Cry3Bb1 selection and backcrossing of the selected strain to the susceptible strain were also performed by Hoffmann *et al.* [34]. Nevertheless, as already mentioned, in the absence of sizeable fitness costs, resistance is expected to endure once it has developed [14,34,44].

The longevity of females from the resistant and susceptible strains reared on three non-*Bt* maize hybrids in the Hoffmann *et al.* [34] study averaged between approximately 35 and 65 days, more than two months less than the mean longevity of 127 (range 7–241) days observed here for the four crosses. This longevity difference between the studies was paralleled by a difference in female fecundity, which averaged 412 and 538 for susceptible and resistant females, respectively, in the Hoffman *et al.* [34] study and 1718 for our four treatments, which did not vary significantly but displayed an opposite tendency toward lower fecundity in resistant females. Our relatively long lifespan and associated high fecundity may have resulted in part because the females in our study were individually isolated except during courtship and copulation, whereas those in the former study were held in mixed-sex groups until six days after emergence and then maintained in mixed-sex pairs during oviposition. Housing females with males is known in *Drosophila melanogaster* (Meigen) [57] to reduce female longevity in part because of prolonged male courtship and repeated mating attempts. Similarly, in the seed beetle *Callosobruchus maculatus* (Fabricius), housing females with males during a mating refractory period following the females’ first mating reduced female longevity and especially fecundity because of male sexual harassment of the females [60].

Most other studies reporting the longevity and fecundity of *D. v. virgifera* adult females [53,61,62] used experimental insects maintained in mixed-sex groups during at least the pre-oviposition period, if not during the oviposition period, and reported longevities and fecundities closer to those of the Hoffmann [34] study than to those of our study. Another factor shown to affect female longevity in *D. virgifera* and *D. barberi* besides their maintenance in mixed-sex groups is their diet [63,64,65]. Nevertheless, similar to our study, Hoffmann *et al.* [34] fed their beetles artificial diet, as did Meihls *et al.* [16], who reported a female longevity of approximately 70 days, on average, as well as differing fecundities of 410 and 570 eggs, respectively, for laboratory-selected Cry3Bb1-resistant and control populations reared as larvae on non-*Bt* maize.

In insects, larger males generally have a mating advantage over smaller males, often through direct competition for mates or through females preferentially mating with larger males [35,40,41,66,67]. However, our no-choice trials conducted using males and females of randomly chosen body sizes detected no differences in the weight of males that mated successfully and those that did not mate, a result similar to that obtained using mixed-sex pairs of *D. barberi* [51]. This result is consistent with the supposition that female *D. v. virgifera* do not discriminate against males based on low body weight, similar to certain other chrysomelids, including the Eucalyptus leaf beetle, *Chrysophtharta agricola* (Chapuis), and the leaf beetle *Oreina cacaliae* (Schrank) [68,69]. In *Oreina gloriosa* F., however, larger males display a mating advantage over smaller males [68]. Unlike the results in the above no-choice tests, French and Hammack [70] showed a large-male mating advantage in *D. barberi* when virgin females were provided a choice between potentially competing large and small males. Another study found positive assortative mating for this species based on male and female body weights [71]. Although choice experiments in which females can select between competing mating partners remain to be reported for *D. v. virgifera*, our current study does suggest the occurrence of moderately strong, positive assortative mating that is predicated on male and female body weights. Kang and Krupke [72] previously reported weight-based, positive assortative mating in *D. v. virgifera* mating pairs at one field location but not at another, implying that selection intensities on body size may fluctuate under field conditions, as reported for the male redback spider *Latrodectus hasselti* Thorell [73,74].

Fifty-eight percent of our females (14% were not mounted) did not mate, a figure close to the 54% reported by Sherwood and Levine [75] for female *D. v. virgifera* first presented with a male and the 45%–48% found for mixed-sex, *D. barberi* pairs [51,71] This mating reluctance could suggest the existence of unidentified male traits important for successful copulation but also most likely includes a component attributable to female receptivity. Using virgin *D. v. virgifera*, Hammack [49] reported that 42% of mixed-sex pairs failed to initiate copulation within two hours; however, that value was 26% or 64% depending on whether the females had called to attract sexually mature males (displayed sex-pheromone-releasing behavior) or not called, respectively, during a 2.5-h period just preceding pairing, with pairing beginning four to seven hours after female emergence from the soil. Regarding male traits important for successful copulation, in the related species *Diabrotica undecimpunctata howardi* Barber, Tallamy *et al.* [47,48] demonstrated the existence of cryptic female choice in that females may copulate with as many as 15 males before accepting a spermatophore, with successful spermatophore transfer correlated with a high rate of stroking of the female by the mounted male using his antennae. In addition, many insects including *D. u. howardi* females use body odor or cuticular hydrocarbons to distinguish suitable mates [39,76,77,78]. For *D. v. virgifera*, the role of cuticular hydrocarbons in mate selection remains to be elucidated; however, Murphy [79] reported qualitative differences in cuticular hydrocarbons between *D. v. virgifera* males developing on refuge (non-*Bt*) maize compared with Cry3Bb1 *Bt* maize. These differences could provide a basis for the identification by a female of Cry3Bb1 resistant males that could offer survival benefits to those of her offspring exposed to *Bt* maize.

Some insects may discriminate against males by extending courtship and/or copulation durations, which potentially could provide additional time for more appropriate mates to displace less suitable ones or for females to assess the genetic quality of their mates [39,48]. Courtship behaviors such as titillating females with their antennae, legs, or other body parts, as discussed above for *D. u. howardi*, also could affect courtship durations and ultimately male mating success [47,80,81,82]. Using these stimulation techniques to coerce females into mating could prolong the courtship of indecisive females. For *D. v. virgifera*, Lew and Ball [81] found that after mounting, males courted females for 10 to 60 min prior to mating*.* In our study, courtship duration ranged from 0 to 118 min but did not vary by genotype. For the copulation duration following courtship, Lew and Ball [81,83] reported an interval of three to four hours in *D. v. virgifera*. However, Kang and Krupke [84] reported that the copulation duration of previously mated *D. v. virgifera* males averaged six hours but could range from three to 33 h. In our study, there were no differences among the four crosses in the copulation duration of mated pairs, which averaged close to three hours overall and ranged from approximately two to five hours, intervals consistent with maximal insemination [75].

The majority of available studies, including our study, report few fitness costs associated with Cry3Bb1 resistance or even with the survival of resistant *D. v. virgifera* on maize producing single *Bt* toxins [16,31,32,33,34]. However, susceptible adults emerging from non-*Bt* maize refuges and able to mate with resistant insects surviving on *Bt* maize, as designed for resistance management, may suffer fitness costs from feeding on above-ground plant tissues that adults use as food, such as leaves, silks, and pollen, because these tissues also express the *Bt* toxin, albeit at a lower dose than that of maize roots [85]. Although Al-Deeb and Wilde [86] and Nowatzki *et al.* [87] found little to no effect on adult body size, longevity, or fecundity, Meissle *et al.* [88] showed that adult feeding by susceptible *D. v. virgifera* on maize tissue from Cry3Bb1-expressing compared with isoline non-*Bt* maize could, depending on the tissue, increase male mortality and reduce female body weight and fecundity. Such feeding on *Bt* maize by susceptible beetles could potentially affect their mating behavior. For example, a reduction in the vigor with which susceptible males court females could result from their ingestion of *Bt* toxins as adults and significantly reduce their mating success in favor of *Bt*-resistant males. The importance of any such fitness costs may increase as maize plants producing higher doses of toxins become more prevalent in the landscape. Experiments on *D. v. virgifera* reproductive behavior and biology similar to those reported here are planned using insects fed as adults on *Bt*-containing diets, as well as under conditions that simultaneously provide a female with more than one potential mating partner.

## 5. Conclusions

We found little impact of Cry3Bb1 resistance under no-choice mating conditions on mating success, mating behavior, fecundity, fertility, and longevity among the two homozygous and two heterozygous crosses between lines resistant and susceptible to the Cry3Bb1 protein. Nevertheless, female longevity did vary with male genotype and was reduced by mating with resistant compared with susceptible males. With higher dose toxins, this pattern could be highly favorable for resistance management should the reduced longevity correlate with reduced fitness. However, females also tended to mate with males of similar body weights, which could promote mating between like genotypes should body size also be subject to selection during exposure to high-dose toxins. Rearing and testing beetles under more natural conditions could reveal subtle differences in their reproductive biology that could help explain the rapid expansion of Cry3Bb1 resistance and facilitate the development of new strategies to prevent resistance to other, more potent toxins.

## Appendix

**Table A1:** Pupal, adult, and differences in weights (mg) following metamorphosis by sex for laboratory-reared and field-collected *Diabrotica v. virgifera*.

Colony ^a^	Sex	*n*	Pupae	Adult	Difference	*r* ^b^
			Mean ± SD	Mean ± SD	Mean ± SD	
Lab (D)	Male	261	11.4 ± 1.8	9.6 ± 1.9	−1.8 ± 1.0	0.84
	Female	298	12.9 ± 2.1	11.1 ± 2.0	−1.7 ± 1.1	0.87
Lab (ND)	Male	301	13.7 ± 2.7	12.2 ± 2.3	−1.6 ± 1.1	0.91
	Female	322	16.2 ± 2.8	14.4 ± 2.5	−1.8 ± 1.4	0.85
Field	Male	164	10.2 ± 2.2	8.7 ± 2.0	−1.4 ± 0.8	0.92
	Female	325	10.5 ± 2.1	9.1 ± 1.9	−1.5 ± 0.8	0.92

^a^ D = diapausing, ND = non-diapausing; ^b^ Spearman’s rho, *p* < 0.0001.
